# Dementia care in Israel: top down and bottom up processes

**DOI:** 10.1186/s13584-019-0290-z

**Published:** 2019-02-20

**Authors:** Netta Bentur, Shelley A. Sternberg

**Affiliations:** 1 Stanley Steyer School for Health Professionals, Faculty of Medicine, Tel-Aviv University, Tel Aviv-Yafo, Israel; 2 Geriatric Devision, Ministry of Health, Jerusalem, Israel

**Keywords:** Dementia, Services development, Implementation

## Abstract

Dementia is one of the main causes of disability among older adults and is viewed as one of the most distressing and devastating of conditions. Dementia has a profound impact on those who suffer from the disease and on their family caregivers. In this article, we describe the added benefit of implementing top-down and bottom-up strategies in the process of influencing and developing healthcare services. We use Israel as an example to argue that breakthroughs in care implementation and development of services are more likely to occur when there is a convergence of top-down and bottom-up processes. In the first section of the article, we present the top-down plans, initiated to address the needs of people with dementia and their families. In the second section, we present examples of bottom-up projects that developed in Israel before and after the top-down plans were initiated. In the third section, we contend that it is the combination of these top-down and bottom-up strategies that led to a breakthrough and the expansion of services for people with dementia and their families, and we argue that the Israeli case study is applicable to other health systems.

## Background

The aging population creates multiple challenges for society, one of the most significant being the need to cope with the steadily increasing number of older adults with dementia. This group of illnesses is one of the main causes of disability among older adults [1] and is viewed as one of the most distressing and devastating of conditions. Dementia has a profound impact on those who suffer from the disease and on their family caregivers. It also has a significant financial impact on society and poses challenges to health and social services [2–4].

According to reliable estimates, approximately 154,000 individuals in Israel have some form of dementia and the figure is expected to rise to around 290,000 by 2030, in tandem with the expected increase in the number of older adults [5, 6]. According to estimates, based on a national survey conducted in Israel more than a decade ago, 84% of people with dementia were living in the community [5, 6]. According to this national survey, 46% of dementia patients living in the community have mild dementia, 23% have moderate dementia and 31% have advanced dementia [7].

Ninety-seven percent of Israelis aged 65+ live in the community [7], including those with dementia (though no data are available on the prevalence of those with dementia). Thus, unlike other Western countries, most of the older adults with dementia live in the community [7]. For instance, 2011 OECD data on long-term care provision (not exclusively for those with dementia) show that 62% of long-term care in the US is provided in the community, compared with 32% in the Netherlands and 91% in Israel [1]. Most of these people with dementia are cared for by family members who provide personal, nursing and medical care for which they receive no financial remuneration. This places a heavy burden – physical, mental and financial – on caregivers at every stage of the illness, and particularly in the advanced stages and at the end of life. As a result of the significant impact of dementia, there is broad understanding in Israel, as worldwide, that there is a need for a systemic response to the consequences of the disease, and practically, for the development of services for older people with dementia and their family members [8].

Researchers and stakeholders have identified two main strategic tracks in the process of influencing and developing healthcare services. In the so-called top-down track, change is stimulated by policy initiatives, i.e., through legislation and regulation, consensus conferences, white papers and directives from directors of health agencies and HMOs (health funds, named as Kupot Holim in Israel). Work plans, goal-oriented activity and projects are then based on these top-down decisions. Budgets may or may not be allocated for these new initiatives. The second strategy, the so-called bottom-up track, promotes change initiated by professionals in the field [9–11]. Working alone or with colleagues, they are the driving force behind interventions that they themselves plan and implement. Many of these initiatives start as local or regional grassroots plans that grow out of professional insights, clinical experience and knowledge accumulated over the years. Some of them start as an initiative of patients or family caregivers who organize to achieve their unmet needs. Compared with government regulations and legislation, which are usually imposed from above, the bottom-up approach makes it possible to adapt an intervention model to the population for whom it is intended [12]. Bottom-up programs can be more flexible, learning from other similar programs and adjusting their models as they go along. Usually they are not bound by strict standardized forms and instruments. As such, professionals can formulate a project in supportive, less threatening, and easily understandable language and make cultural and linguistic adjustments [13].

In this article, we demonstrate the added benefit of implementing top-down and bottom-up strategies together. We use Israel as an example to argue that breakthroughs in care implementation and development of services are more likely to occur where there is a convergence of top-down and bottom-up processes. In the first section of the article, we present the top-down plans that were initiated to address the needs of people with dementia and their families. In the second section, we present examples of bottom-up projects that developed in Israel before and after the top-down plans were initiated. In the third section, we contend that it is the combination of the top-down and bottom-up strategies that led to a breakthrough and the expansion of services for people with dementia and their families, and we argue that the Israeli case study is applicable to other health systems.

## Main text

### Top-down processes

The 2014 Glasgow Declaration called for the creation of a European Dementia Strategy and national strategies in every country in Europe, and called upon world leaders to recognize dementia as a public health priority and to develop a global action plan on dementia [14]. Indeed, many countries have developed national strategic plans to addressing dementia, and in 2013, Israel joined these countries by developing a national strategic plan for dealing with the challenges posed by the disease (the Plan). The necessity of the a program was expressed in Israel by the Geriatric national council, health and social policy makers, stakeholders and services providers, stating that it is essential in order to comprehensive identification and expressing the needs and developing a coordinated respond to the present and future requirements in Israel.

The Plan was formulated by an interdisciplinary group of experts from government ministries, the National Insurance Institute, the HMOs, which are responsible for providing health services in the community, hospitals, nonprofit organizations and leading academics. The process was led by the Center for Research on Aging at the Myers-JDC Brookdale Institute and the National Geriatric Council, with the support of the Helen Bader Foundation. In order to prepare the plan, leading experienced researchers from the Myers-JDC-Brookdale institute gathered epidemiological and demographic data that was available in Israel about people with dementia in the community and in long term care institution. They also gathered information regarding all the health and social services that are currently available for people with dementia and for their caregivers. The researchers firstly brought the issue of dementia to the table in 2005, and since then have conducted two studies that produced a huge bulk of detailed information about the unmet needs of the population with dementia and their families. The researchers also held interviews with leading Israeli health and social policy makers and service providers [15, 16]. The product of all this work was a comprehensive booklet of data and information that served the forum of experts in their work.

The Plan’s vision is to enable people with dementia and their families – through a broad array of easily accessible, high-standard supports and services – to live as full, independent and dignified a life as possible. The Plan recognizes the need to address dementia from the stages of prevention of risk factors and early detection, through diagnosis and patient care, to the assurance of quality of end-of-life care. It proposes to do so by implementing interdisciplinary work principles and care, and by encouraging coordination and cooperation between all participating parties.

In the first stage of preparing the Plan, the interdisciplinary group conducted a comprehensive review of the current status of dementia care in Israel and identified the major gaps and challenges that needed to be addressed.

It was found that there was a lack of awareness of health and social services available for the prevention, diagnosis, and treatment of dementia, and support for people with dementia. This lack of awareness led to under-diagnosis, limited early intervention, ineffective care management, stigmatization of people with dementia, and a lack of suitable family support. It was also found that there was not enough training and professional knowledge in the field of dementia. In addition, although there was broad consensus that people with dementia should be able to continue to live at home as long as possible, there was a need to ensure the availability of quality institutional care for those that can’t stay at home. Family caregivers of people with dementia were also identified as a target population with a lot of unmet needs, and therefore requiring the development of services in order to address their concerns and ensure their physical and mental health.

Based on this review, the group formulated a set of objectives for the strategic Plan:

1. Raising public awareness of dementia and dispelling the associated stigma
2. Improving the array of community health services offered to provide comprehensive care at every stage of the illness
3. Improving the array of community social services (such as those provided through the Long-Term Care Insurance Law and daycare centers)
4. Developing interventions to support family caregivers directly
5. Adapting the array of long-term institutional services to meet changing needs
6. Developing and expanding resources to train care professionals in the community and in hospitals
7. Promoting critical research to support policy planning and service development.

The development of the Plan followed other processes to improve the care of people with dementia that had taken place in Israel a few years previously. The most significant was the formulation of clinical guidelines by a clinical consensus conference held in November 2011, initiated by the Israeli Medical Association and adopted by the Ministry of Health. The clinical guidelines included four recommendations. The first was for the clinical evaluation of dementia by a multi-professional team, using psychometrical and performance tests. The second was to prevent dementia by conducting cardio-cerebro-vascular risk tests, promoting Mediterranean nutrition and physical activity. The third and fourth recommendations referred to medical and non-medical therapy such as medication, cognitive training, behavioral intervention and recreation therapy.

Four years before this consensus conference, the medical administration at the Ministry of Health issued a directive to the HMOs requiring them to develop comprehensive geriatric evaluation clinics in the community (12/2007). One of the main tasks of these clinics was the obligation to undertake cognitive evaluation, using shared objective tools. Indeed, a study from 2011 found that about half of the older adults who were referred to comprehensive geriatric assessment did suffer from cognitive decline and that one of the main benefits of the clinics for family physicians was helping them to treat patients with cognitive deterioration [17].

Another top-down indirect action that should be mentioned is a policy statement on palliative care, issued by the Ministry of Health director-general in 2009, describing the minimal standards for the development of palliative care services by all hospitals and by the four HMOs. Besides being an important development in itself, this was the first time in Israel that the Ministry of Health mandated palliative services for people with advance dementia and not only for cancer patients.

### Bottom up activities

It is incorrect to state that there were no activities or services for people with dementia and their families before the development of the Plan. The first bottom-up organization was Melabev (the Hebrew acronym for Center for Treatment of older adults in the Community), a nonprofit organization, that was established in 1981 by Leah Abramowitz, a social worker, and Prof. Arnold Rosin, a geriatrician, with their colleagues at the Shaare Zedek Medical Center in Jerusalem. They witnessed the problems faced after diagnosis by patients and their families, who lacked advice and facilities for coping. They established a geriatric institute in partnership with Shaare Zedek Medical Center to offer the first in-service courses in Israel for eldercare professionals. Over the years, Melabev has grown, with the mission to enhance quality of life with dignity and self-worth in adults who are affected by dementia or Alzheimer’s, to help slow their cognitive and physical decline through specifically designed therapeutic and physical activities allowing patients to maintain their current level of functioning for as long as possible. After more than thirty years of activity, the organization provides multiple services for people with dementia, although the main center of their activities is in Jerusalem.

At the same time, as Israel’s population began to shift from a very young composition to an older composition, it was brought about a realization of the urgent need for community-based services for this rapidly growing older fragile adult population. One of the key services developed by Eshel (The Association for the Planning and Development of Services for the Aged in Israel, that is the biggest Israeli NGO that develops services for older adults) was day centers for older adults, which became a central component of the national system of community-based care. Prior to this initiative, day care centers was virtually unknown in Israe. The idea was first raised by the Ministry for Social Affairs during the early eighties of the 20th cnturey and was implemented by Eshel. Since the mid-1990s, the service has developed at an accelerated pace and today 174 day centers exist nationwide. They provide socio-cultural activities, personal-care services, meals, and professional therapeutic services – all under one roof. Day care centers are part of the basket of services offered to older adults eligible under the Community Long-term Care Insurance Law. Daycare centers are common response for mentally-frail elders living in the community. They offer elders occupation and activities, and their families – a respite. About a third of all daycare centers have a special department for the mentally frail older adults with dementia or have a separate wing for this population [18].

Another longstanding bottom-up organization is the Alzheimer’s Association of Israel (EMDA), which was founded in 1988 by family members of people with Alzheimer’s and other form of dementia. It is another nonprofit organization, which was founded to support the patients’ needs and help them through stressful times. Although for the first two decades after its founding, EMDA was a well-known but relatively small organization that operated mainly in the Tel-Aviv area, over the past few years, particularly since the introduction of the Plan, EMDA has expanded extensively and now has 38 branches nationwide. Its growth has included the expansion of current activities as well as the creation of new ones.

Several other non-governmental organizations, such as Yad Sarah and Ezer Mizion, have also developed services for older people with dementia and their families over the last two decades. Both organizations were set up by their founders in memory of their late patents who suffered from degenerative diseases and disabilities, with the mission to keep older people in their homes and out of institutions as long as possible. These organizations supply a variety of services to people in the community, including centers for Alzheimer’s, that offer counseling and ongoing personal guidance to families of dementia patients.

Although Israel had an extensive primary healthcare system in the community provided by the four HMOs, they lacked targeted interventions to improve the care of people with dementia. Nevertheless, some activities deserve mention. In 2016, Clalit Health Services, the largest HMO in Israel, which provides medical care to 55% of the Israeli population, has introduced the Five Wishes program, which was originally introduced in the US in 1996 (https://www.agingwithdignity.org/five-wishes/about-five-wishes). It is an easy-to-use document written in everyday language, which allows people to plan how they want to be cared for in the event of serious illness and inability to speak for themselves. The Five Wishes is considered a short and effective tool that facilitates discussion with people who are not necessarily in a terminal condition, during a routine visit to the family physician or community nurse. This approach is unique because it addresses medical, personal, emotional and spiritual needs, and helps structure discussions between patients, their families and physicians. Currently, the tool is being distributed among all of Clalit’s primary care providers (physicians and nurses) throughout Israel, but there is no information yet about the extent of its use.

In addition, Maccabi Healthcare Services, the second largest HMO plan in the country that provides medical care to about 25% of the Israeli population, initiated a pilot project to provide home hospice care for advanced dementia patients. This pilot project showed a decrease in hospitalizations and an increase in family caregiver satisfaction with care, and resulted in a change of policy in the HMO. (see article by Sternberg et al. in this journal).

### The convergence of top-down and bottom-up processes

Since its launching 5 years ago, the National Strategic Dementia Plan has been promoted many initiatives. This could not have been achieved without the grassroots organizations that were already in the field and served as a solid basis for development and expansion. Thus, for example, ESHEL and EMDA provided a service infrastructure that the program joined, which enabled the development of new services. Moreover, the existing services provided the most important resource, i.e. professionals with extensive knowledge and experience in development of services for people with dementia and their families. They all supported the National Program and willingly became part of their work. The knowledge, experience and long-term involvement of these professionals had a huge impact on developing programs.

The annual budget for the program, through partnerships, has increased from under 1 million NIS in the first year (2013–2014) to close to 10 million NIS in 2017.

The Ministry of Health is leading the Plan through an implementation committee of stakeholders form all relevant organizations, and promotes the development of models of care through demonstration projects that are evaluated by accompanying research studies. The goal of one of the first projects, started in 2014, was to improve the diagnosis and treatment of dementia in primary care. Each of the four HMOs designed its own project, which included a program to train nurses who are responsible for the care of people with diabetes, to identify cognitive impairment, a cell phone application to help primary care doctors to do a mini geriatric assessment, development of a dementia registry, and training sessions for multidisciplinary staff. Another project in development, aims to improve the continuity of care for people with dementia or delirium as they transition from the community to the acute care hospital and back into the community.

Another project started in 2017 in four general hospitals, aims to improve the diagnosis, treatment & discharge of inpatients with dementia or delirium. Each hospital developed its own project which include improving the care of people with dementia who come to the emergency room after trauma, and training volunteers to work with hospitalized patients with dementia based on the American model called HELP (https://www.agingwithdignity.org/five-wishes/about-five-wishes), to decrease the incidence of delirium .

On the social front, EMDA has implemented a Dementia Friends program [19] in partnership with local municipalities and has trained 6000 volunteers so far. This British program aims to raise public awareness to the needs of people with dementia and their families. The Dementia Friends program recruits volunteers who learn about the disease and engage in different activities in their communities in order to tackle the stigma and lack of understanding that leaves many people lonely and socially excluded.

In addition, caregiver support programs have been developed including 68 caregiver support groups of EMDA and 25 personalized groups, with the collaboration of the National Insurance Institute, ESHEL and EMDA. In addition, respite care expanded to 33 nursing homes nationally, with the collaboration of the Ministry of Health and the Welfare Ministry.

The Israel National Dementia Plan has engaged major stakeholders, and has generated effective partnerships projects. Funding has gradually expanded from the Minstry of Health, ESHEL,, and from the National Insurance Institute. Preliminary data from the pilot projects evaluations are currently attained and analysed. Following the results of these evaluation, the head of the plan at the ministry of health together with the implementation committee will design in the coming months a scheme for applying some of the projects as standard of care at the HMOs and for recommending changes in other projects.

## Conclusions

Providing quality services for people with dementia and their families is a challenging and complex task, but it can be done. In this article, we argue that parallel top-down and bottom-up processes should be initiated within the health and social systems. A comprehensive national strategic plan that was formulated by a group of experts cannot bring about change by itself. It may be the first important step and a basis for an agreed-upon policy, but more is required if the policy is to be implemented and incorporated into practice [20]. The convergence of both strategies creates a new field of activity and contributes far more than either strategy alone in addressing the challenges of this complex and change-resistant area. It is the synergy between top-down and bottom-up activities that is addressing the challenges of improving the access to quality dementia care.

The major initiatives of the plan have led to increased awareness, improved care and access to medical and social services, and work force expansion and training. However, although the enormous development of services for people with dementia and their families is impressive, much more still needs to be done. Since most Israeli older adults live in the community, more effort and resources should be allocated to community health services. Studies show that older adults with dementia and their family members have reported unmet needs and suffering (https://www.dementiafriends.org.uk/). For example, there is a need to improve the education of family physicians in the early detection of dementia and the assessment of cognitive and behavioral deterioration. There is also a need for much more comprehensive geriatric evaluation clinics and home care for people with dementia. Community-based palliative care for dementia needs to be developed since studies have shown that people with advanced dementia suffer from pain, skin breakdown and other symptoms [17, 21]. It is anticipated that the convergence of a top-down directive, and an increase in bottom-up programs will lead to the development of better community – based palliative care for dementia. At the same time, studies are needed to assess the quality of these programs to stimulate new bottom-up initiatives and facilitate decisions for future planning.

Because Israel is a small country with a relatively uniform health system, it can serve as a case study of using the top-down and bottom up approach. This case study may be applicable to other countries by reinforcing the importance of parallel development of top-down and bottom-up initiatives. The question of where to start is less important than the understanding that parallel and synergistic initiatives are imperative. Much more needs to be done, but the top down and bottom up convergence of strategies and initiatives has created stakeholders in dementia care. The Israel National Dementia Plan has become a model for other Israeli national initiatives in palliative care and fall prevention.

## Abbreviation

EMDA: Alzheimer’s Association of Israel
